# The fitness trade-off between growth and stress resistance determines the phenotypic landscape

**DOI:** 10.1186/s12915-024-01856-7

**Published:** 2024-03-13

**Authors:** Dongsan Kim, Chae Young Hwang, Kwang-Hyun Cho

**Affiliations:** Department of Bio and Brain Engineering, Korea Advanced Institute of Science and Technology (KAIST), 291 Daehak-Ro, Yuseong-Gu, Daejeon, 34141 Republic of Korea

**Keywords:** Fitness trade-off, Growth, Survival, Genotype, Phenotype

## Abstract

**Background:**

A central challenge in biology is to discover a principle that determines individual phenotypic differences within a species. The growth rate is particularly important for a unicellular organism, and the growth rate under a certain condition is negatively associated with that of another condition, termed fitness trade-off. Therefore, there should exist a common molecular mechanism that regulates multiple growth rates under various conditions, but most studies so far have focused on discovering those genes associated with growth rates under a specific condition.

**Results:**

In this study, we found that there exists a recurrent gene expression signature whose expression levels are related to the fitness trade-off between growth preference and stress resistance across various yeast strains and multiple conditions. We further found that the genomic variation of stress-response, ribosomal, and cell cycle regulators are potential causal genes that determine the sensitivity between growth and survival. Intriguingly, we further observed that the same principle holds for human cells using anticancer drug sensitivities across multiple cancer cell lines.

**Conclusions:**

Together, we suggest that the fitness trade-off is an evolutionary trait that determines individual growth phenotype within a species. By using this trait, we can possibly overcome anticancer drug resistance in cancer cells.

**Supplementary Information:**

The online version contains supplementary material available at 10.1186/s12915-024-01856-7.

## Background

One of the unsolved central questions in biology is identifying the genomic and molecular sources of phenotypic variations [1]. For *Saccharomyces cerevisiae*, a number of studies have been conducted to identify the genomic loci that are associated with particular phenotypes such as gene expression levels or growth rates [2–5]. Among many phenotypic outcomes, the growth rate is of importance as a central phenotype for a unicellular organism. Within yeast populations, natural variants have a number of polymorphisms including single nucleotide polymorphism, insertion and deletions, and copy number variations that result in differences in molecular functions [5]; therefore, each natural variant has a different growth rate depending on the respective environmental condition. The relationship between genomic variation and the growth rate under a certain environmental condition is complicated, as it relies on a huge number of genomic diversities [3]. Despite the inherent complexity, it has been known that most genomic perturbations or variations are not impactful to phenotypes (robustness), and only a few genetic perturbations dominate phenotypic variations (fragility) [6]. Meanwhile, a cellular system cannot obtain maximal fitness for every environmental condition it faces, referred to as a fitness trade-off between growth and survival [7, 8] (i.e., a cell with a high growth rate in one condition shows a low growth rate in another condition). The existence of robustness and fragility, and fitness trade-off suggest the possibility for the existence of key genetic components that regulate numerous phenotypes.

It has been widely studied that a yeast strain with higher growth in rich condition provides worse growth in nutrient-limiting conditions [9] or stress conditions [10]. Wenger et al. [9] further found that a yeast strain evolved high fitness in a certain nutrient-limiting condition and could also show enhanced fitness in other such conditions yet displays reduced fitness in rich conditions. Moreover, Zakrzewska et al. [10] found that a yeast strain that gained stress tolerance in one condition also can acquire tolerance in other stressful conditions, suggesting a tight coupling between general stress response and cellular growth rate [11]. However, these studies were mostly based on laboratory yeast strains with genetic modifications, and an analysis exploring the relationship from genotype and gene expression profiles to fitness trade-offs across a diverse range of yeast strains has not yet been conducted.

Here, we investigated common genetic variants that regulate multiple growth phenotypes from diverse natural yeast isolates to the genetically crossed segregants of laboratory (BY) and wine (RM) yeast strains. By analyzing the association of growth phenotypes across various conditions, we found that all yeast isolates extensively show fitness trade-off between two clustered conditions. Based on transcriptomic analysis, we also identified that yeast strains share two gene sets showing mutually exclusive expression pattern with different functionality, indicating that these gene sets underlie the molecular mechanism behind the fitness trade-off. Furthermore, from quantitative trait loci (QTL) and genome-wide association (GWA) analysis for the growth phenotypes, we found potential regulators that are crucial for the fitness trade-off. By analyzing anticancer drug resistance profiles, we found that the fitness trade-off in yeast also accounts for the drug resistance of various cancer cell lines. By applying this finding, it was possible to induce the sensitivity of the cancer cells to anticancer drugs, regardless of different mutational backgrounds, cell types, and anti-cancer drugs. Taken together, we suggest that the fitness trade-off is an underlying principle that determines the individual phenotypic variation within a species.

## Results

### Finding the fitness trade-off from the yeast growth phenome

**Fig. 1:**
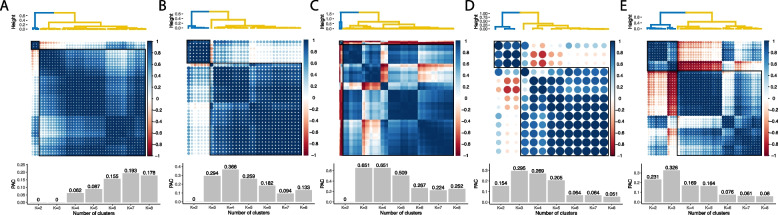
The fitness trade-off under various environmental conditions. Hierarchical clustering on the growth phenotypes across multiple conditions, the corresponding correlation plot, and the PAC measurements for each cluster. **A** denotes the growth profiles from 45 nutritional conditions of 1011 natural yeast strains. **B** indicates the growth profiles across 35 stress conditions of 975 natural yeast strains. **C** indicates the growth profiles for 38 genetically diverse yeast strains from 199 conditions. **D** denotes 13 stress conditions of 52 genetically diverse yeast strains. **E** denotes the growth profiles for 1008 segregants from a cross between BY and RM strains. We performed hierarchical clustering between the conditions using 1 − Spearman correlation as distance with the average linkage method. We note that the conditions are robustly clustered into two groups (as shown by two black boxes in each correlation plot and PAC index). In **A**, both 2 and 3 are the optimal number of clusters. In **B** and **C**, 2 is the optimal number of clusters. In **D**, 2 is the optimal number of clusters without singletons in the cluster (*K* < 6). **E** is the only phenotype that 2 is not the optimal number of clusters. In the presented analysis, dot sizes are proportional to the magnitude of the Spearman correlation coefficients, with larger dots indicating stronger absolute correlations

We collected the growth rates of diverse natural isolates as well as the progeny of a large cross between two representative yeast strains (BY and RM) across various environmental conditions from five independent studies (see the “Methods” section). After normalizing and obtaining reconstructed growth phenotypes of low rank (see the “Methods” section), we computed the correlation of the reconstructed growth phenotypes across multiple conditions. We found that the environmental conditions can be divided into two groups for most datasets analyzed, showing a common tendency of similar growth rates within clusters and antagonistic growth rates between the groups (Fig. 1 and Additional file 1: Fig. S1). We further investigated whether this tendency remains consistent for each yeast clade class (wild, domesticated, or unassigned class) or different stress types (nutrient requirements, environmental and metabolite stresses or toxins). We found the same tendency for domesticated and unassigned classes but not wild classes (Additional file 1: Fig. S2 and S3). We also found that the tendency to form two clustered phenotypes largely remains regardless of stress types (Additional file 1: Fig. S4). These results indicate that domesticated (and unassigned) yeast strains exhibit a clear fitness trade-off against various general environmental conditions, while wild yeast strains relatively do not, implying that domestication events drive yeast to evolve dichotomous growth phenotypes to a diverse range of environments.

**Fig. 2:**
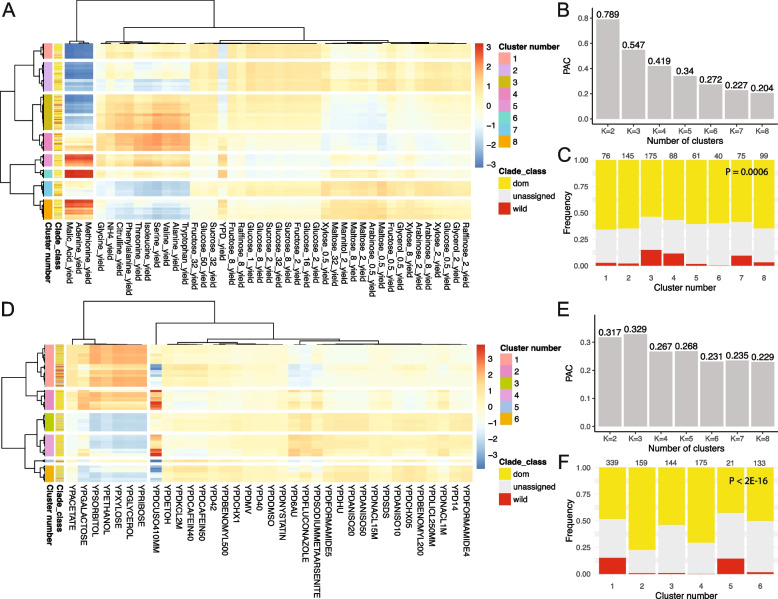
Clustering of growth phenotypes in yeast strains under nutritional and stress conditions. **A** A heatmap represents growth phenotypes, with each row corresponding to a nutritional condition and each column to a yeast strain. Strains are categorized as “wild type,” “domesticated (dom),” or “unassigned.” Color reflects the growth phenotype, with values scaled by row. Side color bars indicate cluster numbers and clade classes. Hierarchical clustering analysis was performed for each row and column using 1 − the Spearman rank correlation coefficient as the distance metric and the average linkage method for clustering. **B** Eight yeast strain clusters were determined based on the PAC. The dendrogram across conditions is identical to that in Fig. 1A. **C** A stacked bar plot shows the frequencies of the three yeast clade classes across each cluster, with wild strains enriched in specific clusters. For panels **D**–**F**, an identical analysis was conducted for stress conditions. The dendrogram across conditions in panel **D** matches that in Fig. 1B. Based on panel **E**, six yeast strain clusters were identified, and a stronger tendency for wild strain enrichment under stress conditions than nutritional conditions was observed. For panels **C** and **F**, the number on each bar indicates the number of yeast strains for each cluster. *p*-values were calculated using a chi-squared test

Next, we investigated whether there are specific yeast clades that show similar growth phenotypes under various nutritional [12] or stress [5] conditions. Wild strains tend to cluster for both datasets, but this tendency is much more significant under stress conditions than various nutritional conditions (Fig. 2A–F). Among stress conditions, wild strains cluster with higher fitness under different energy sources (acetate, galactose, sorbitol, ethanol, xylose, glycerol, and ribose) than glucose as nutrition. Even after excluding the nutrient-limiting condition from our analysis, we observed that wild yeast strains consistently formed a group exhibiting higher fitness under conditions treated with cycloheximide, anisomycin, and caffeine (Additional file 2: Table S1). These results suggest that the environmental stress conditions, particularly starvation conditions, might mirror the adaptive landscape of wild yeast strains. Furthermore, adaptation to one stress condition may confer the ability to withstand other stresses, indicating a mechanism of cross-stress resistance in yeast [11]. On the other hand, we found that under various nutritional conditions indicative of specific yeast habitats, conditions treated with adenine, malic acid, and methionine form a cluster with similar growth phenotypes. However, growth phenotype differences between yeast clades were not observed for these conditions [12]. This suggests that while adaptation to specific habitats may be strain-specific, adaptation to one habitat could confer fitness benefits in others, potentially at the expense of reduced fitness in alternate environments.

### Identifying a recurrent gene expression pattern of the fitness trade-off

We anticipated that there should be underlying molecular characteristics behind the universal fitness trade-off across yeast strains. To investigate this hypothesis, we obtained two classes of datasets: Agilent microarray datasets of various yeast strains under various conditions [13–15] and RNA-seq datasets across natural yeast strains in batch cultures [16]. We also collected microarray datasets from steady-state chemostat cultures that are limited by a variety of natural nutrients but non-stress conditions [17–20]. These datasets were generated to identify the relationship between specific nutrient types or concentrations and the growth rate under non-stress conditions.

**Fig. 3:**
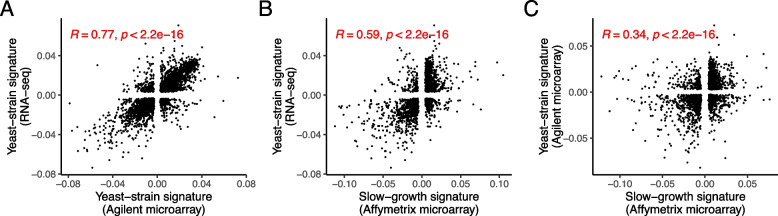
A recurrent gene expression signature across various gene expression profiles of yeast. Scatter plots for the recurrent signatures obtained from the gene expression profiles of yeast. Each dot indicates a gene. Yeast-strain signature (Agilent microarray) denotes the recurrent gene expression signature obtained from gene expression profiles across various yeast strains and conditions. Yeast-strain signature (RNA-seq) indicates the recurrent gene expression signature obtained from various yeast strains measured by RNA sequencing. Slow-growth signature (Affymetrix microarray) denotes the recurrent gene expression signature computed from gene expression profiles of laboratory yeasts under nutrient-limiting but non-stress chemostat culture conditions. *R* and *p*-value denote Spearman’s rank coefficient and the corresponding *p*-value. We note that the recurrent gene expression signature comprises a vector of genes, each with a unique value, denoted as the gene signature score

**Fig. 4:**
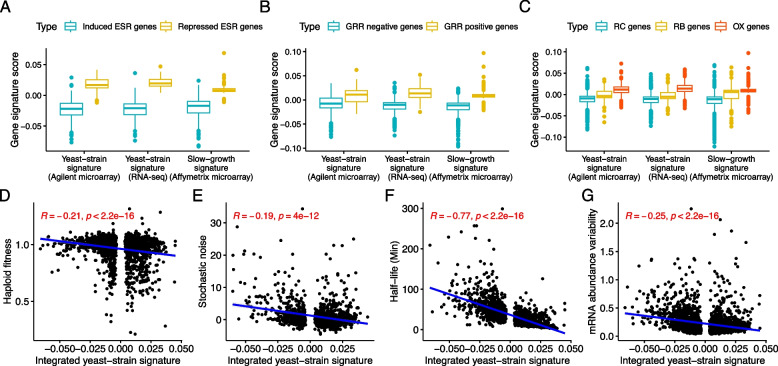
Genomic properties of the genes in the recurrent gene expression signatures. **A**–**C** Boxplots illustrating the distribution of various signature scores for the predefined gene sets. The gene sets are associated with environmental stress response, growth rate response, and metabolic cycle. **D**–**G** Scatter plots between various genomic measurements for each gene and the integrated yeast-strain signature score identified in our study. *R* and *p*-value indicate Spearman’s rank correlation coefficient and the corresponding *p*-value

First, we analyzed mRNA expression profiles across various yeast strains under different conditions. We found that they share a commonality between recurrent gene expression signatures even though the gene expression profiles were obtained from different conditions and genetic backgrounds (Additional file 1: Fig. S5) (see the “Methods” section). We also found that multiple gene expression datasets from continuous cultures share a commonality between the obtained recurrent gene expression signatures (Additional file 1: Fig. S5). Next, we integrated each dataset separately and identified recurrent gene expression signatures. We then compared these signatures across the three datasets to investigate how the recurrent gene expression signatures from diverse yeast strains, termed yeast-strain signatures, differ from those observed in chemostat culture, which represent slow growth without stress in yeast, termed slow-growth signature (see the “Methods” section). We discovered that the yeast-strain signatures are more correlated with each other than with the slow-growth signature (Fig. 3A–C). Next, we examined the distribution of gene signature scores which are the values assigned to each gene within the recurrent gene expression signatures. A gene with a larger gene signature score, whether negative or positive, is considered a more critical determinant in differentiating gene expression profiles between samples. When comparing the distribution of gene signature scores for environmental stress response (ESR) genes [21], we found that the differences between the distributions of ESR-repressed and ESR-induced genes were much larger in the yeast-strain signatures than in the slow-growth signature (Fig. 4A). This finding suggests that, while a slow-growth signature is present to some extent across diverse yeast strains, the inherent differences in cellular states between yeast strains are more closely associated with the yeast stress response. On the other hand, we do not find any significant difference between the yeast-strain signatures and the slow-growth signature for universal growth-rate response (UGRR) genes [22] or the yeast metabolic cycle genes (reductive charging (RC), oxidative (OX), reductive building (RB)) [23] (Fig. 4B and C). These findings indicate that the primary factor driving the differences among yeast strains is their varying sensitivity to general environmental stress responses.

As we confirmed the existence of a commonality between the two yeast-strain signatures, we obtained an integrated, robust yeast-strain signature, then investigated the functional characteristics of the genes in the signature (Additional file 2: Table S2) (see the “Methods” section). Since the gene expression profile is zero-centered scaled, so is the recurrent gene expression signature. Therefore, we divided the integrated yeast-strain signature into two gene sets, having positive or negative scores resulting in 1326 positive-scored genes (PS genes) and 1327 negative-scored genes (NS genes), respectively. We note that those gene sets with the same signs tend to positively co-express whereas those gene sets with the opposite signs favor negative co-expression. Using gene ontology (GO) enrichment analysis [24], we found that the PS and NS genes are mostly involved in “ribonucleoprotein complex biogenesis” and “catabolic process,” respectively (Additional file 2: Table S3).

To investigate the different characteristics between the integrated yeast-strain signature and the slow-growth signature, we obtained PS (or NS) genes in the integrated yeast-strain signature out of NS (or PS) genes in the slow-growth signature, termed yeast-strain specific PS (or NS) genes. We investigated enriched GO terms for the yeast-strain specific PS and NS genes and found that the GO terms “transition metal ion transport” and “proteolysis” are the most significant, respectively (Additional file 2: Table S4). This result suggests that these gene expression programs are unique across yeast strains and deviate from those associated with slow growth in the absence of stress.

We further examined the relationship between the integrated yeast-strain signature scores and various genomic measurements for each gene. Expectedly, the signature score is negatively correlated with yeast haploid fitness [25] (Fig. 4D), indicating that the PS genes are more essential than NS genes for cell growth. We also found that the recurrent gene expression pattern shows a positive correlation with phenotypic potential (Additional file 1: Fig. S6) [26]—a summarizing measurement for multiple quantitative phenotypes (e.g., size and shape) for the knockout of each gene, denoting that the recurrent signature is not only associated with the growth phenotype but also many other quantitative phenotypes of yeast. Regarding gene expression characteristics, the stochastic expression variation [27] for each gene also showed a strong negative correlation with the recurrent gene expression pattern (Fig. 4E). It is also correlated with many of the other gene expression variation measurements, which are interconnected with each other [28] (Additional file 1: Fig. S6). Interestingly, this integrated yeast-strain signature shows a strong association with the half-life of mRNA [29] (Fig. 4F). The integrated yeast-strain signature does not show correlation to the abundance of mRNA (Additional file 1: Fig. S6) [30] but with the mRNA abundance variation [30] (see the “Methods” section) (Fig. 4G). These indicate that the PS genes are tightly regulated in terms of mRNA dynamics; on the other hand, the gene expression of NS genes is highly stochastic depending on conditions and genetic backgrounds, which provides a mechanistic explanation of how yeast, a unicellular system, has evolved to survive and reproduce. Overall, our findings imply that the recurrent gene expression signature forms a molecular basis by which yeast strategically regulates the balance between growth and survival.

### Identifying the genetic components that are associated with the balance between growth and survival

**Fig. 5:**
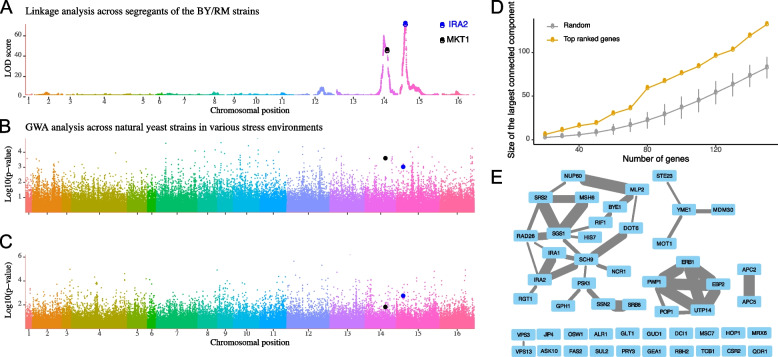
The association between the genotype and the growth phenotype across yeast strains. **A** A LOD score plot illustrating the association between the genotype and the overall growth phenotype of BY and RM segregants. **B**, **C** Manhattan plots depict GWA between the overall growth phenotypes under various nutritional and stress conditions, and the genotypes of different yeast strains. **D** An error bar plot depicts the network size of the top-ranked genes and the random gene samples with the same number. The *x*-axis represents the number of genes analyzed, while the *y*-axis shows the size of the largest connected component in the gene interaction networks. The error bar indicates the standard deviation across 1000 random iterations. **E** An illustration of the identified network for the 50 highest-ranked genes, which were identified by aggregating *p*-values from six GWA analyses that examined stress and nutritional conditions across domesticated, wild, and unassigned yeast strains. The thickness of each edge denotes the confidence of the functional association between two genes. The network is visualized using Cytoscape software [37]

To identify the genetic sources that can potentially regulate the balance between growth and survival across yeast strains, we performed linkage analysis on a large cross between a laboratory yeast strain (BY) and a wine yeast strain (RM) dataset where growth phenotypes and high resolution of locus information are available between yeast strains [11]. To identify the common genetic linkages across 46 different growth phenotypes, we computed the first principal component of the growth phenotypes measured as an overall growth phenotype (see the “Methods” section). Then, we identified QTLs that are associated with the phenotype (see the “Methods” section). Among the studied genes, MKT1 and IRA2 are the most notable in proximity to each respective significant locus, due to their well-established relevance to the QTLs (Fig. 5A). MKT1, a protein similar to nucleases that forms a complex with PBP1 — a poly-A binding protein — and regulates the stability of various mRNAs, is known to be related to a large number of gene expression variations and phenotypic trait differences [14, 31–33]. Another potential causal gene, IRA2 is a GTPase-activating protein that directly interacts with and negatively regulates RAS activity. IRA2 is also known to be associated with multiple gene expression variations as well as phenotypic variations [14, 31]. In particular, IRA2 gene was the most and second most significant locus associated with the gene expression level upon glucose and ethanol treatments, respectively, between BY and RM segregants [14]. Furthermore, IRA2 from the BY strain is known to be less functional than that from the RM strain; therefore, segregants with IRA2 from the BY strain have more active RAS/cAMP/PKA states in comparison to those with IRA2 from the RM strain [14]. Overall, we can summarize that the basal activities of the RAS/cAMP/PKA, which are determined by the genotype of IRA2, regulate many gene expression levels, and hence determine the balance between cellular growth and survival. In other words, a strain with hyperactive RAS/cAMP/PKA states having BY-originated IRA2 shows higher gene expression levels associated with cellular growth (PS genes) but lower gene expression levels associated with cellular survival (NS genes) than that of a strain having RM-originated IRA2. To analyze whether this finding is specific to certain yeast strain segregants (BY/RM), we further performed a GWA analysis using the genotype and the two growth phenotypes from 1011 natural yeast isolates [5, 12] (see the “Methods” section). We again computed the first principal component as a proxy for an overall growth phenotype for each dataset. We were not able to identify any genotype with strong statistical significance (FDR < 0.05) associated with overall growth phenotypes across both datasets indicating that a single gene cannot determine overall growth phenotypes (Fig. 5B and C). For stress conditions [5], TAO3 (*p* = 2E − 05), INO80 (*p* = 3E − 05), and KAR2 (*p* = 4E − 05) were identified as the three genes with the lowest *p*-values. Among these, we note that INO80 plays a role in modulating stress gene transcription, while KAR2 functions as a chaperone to mediate protein folding [34]. For various nutritional conditions [12], MDM30 (*p* = 5E − 06), SRB8 (*p* = 1E − 05), and CSR2 (*p* = 1E − 05) were identified as the three genes with the lowest *p*-values. We also note that MDM30 is known to be associated with mitochondrial fusion, while CSR2 is linked to the utilization of carbon sources [34]. We compared these genes and the GWA hits for specific conditions [5, 12], but the genes were not overlapped, implying the difference between the overall growth phenotypes and the growth phenotype under specific conditions. We further investigated the top-ranked genes, based on *p*-values, by performing GWA analyses for three yeast clades: domesticated, wild, and unassigned (see the “Methods” section). We found that the top-ranked genes do not show any significant overlap between each other in most cases. These findings suggest that the most significant genes vary depending on the conditions and yeast clades (see Additional file 3 for the gene lists and Additional file 2: Table S5 for the GO analysis). However, we found that the top-ranked genes tend to be functionally interconnected, regardless of the differences in growth conditions or yeast clades (Fig. 5D and Additional file 1: Fig. S7) (see the “Methods” section). We have aggregated the *p*-values across conditions for each gene and then obtained the largest connected component based on the ranking of the aggregated *p*-values (Fig. 5E). We could not identify MKT1 as a potential causal gene. However, it is known that a nucleotide variation at the 89th base sequence of the MKT1 gene induces various growth phenotypic changes by regulating sporulation efficiency [35]. Given that the minor allele frequency at this position is less than 5% of the strains investigated [5] and the SNP is very specific to the S288C strain [35], therefore, the growth phenotype alterations attributed to the MKT1 gene can be considered specific to the BY strain, S288C-derived strain, and its segregants. On the other hand, we identified IRA2 gene, along with IRA1 and SCH9 genes, signaling molecules that sense nutrient levels or various stresses as regulators of the RAS/cAMP/PKA pathways [36]. We also found genes (SRS2, SGS1, RAD26, RIF1, and MSH6) that are associated with DNA replication and repair [34]. Intriguingly, we found that the genes in the other connected components are directly associated with ribosomal RNA (PWP1, POP1, UTP14, ERB1, and EBP2) or anaphase-promoting complex of the cell cycle (APC2 and APC5) (Fig. 5E), suggesting that their functional changes can affect the overall growth phenotypes. In summary, these results suggest that the findings from BY/RM segregants do not entirely align with those from various natural yeast isolates, but there is also a commonality that the IRA2 gene is pivotal in the variation of growth phenotypes, through the regulation of RAS/cAMP/PKA state sensitivities. Furthermore, genotypic differences that affect the regulation of ribosomal RNA and the cell cycle contribute to the growth and survival of yeast, thereby contributing to the fitness trade-off.

### The relationship between the growth phenotype of yeast strains and the drug resistance of cancer cells

**Fig. 6:**
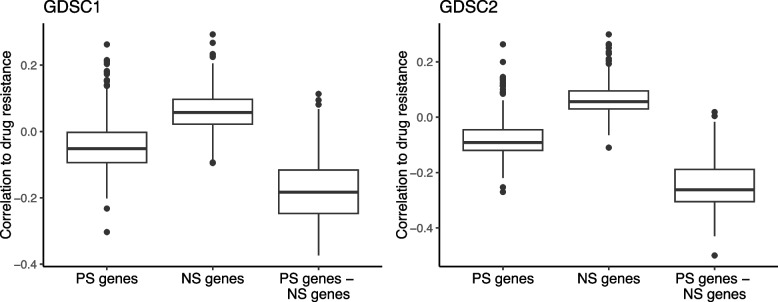
The relationship between drug resistance and the gene expression levels of cancer cells. For each anticancer drug, we computed Spearman’s rank correlation between the median gene expression levels of the PS genes and the drug resistance across cancer cell lines. We also applied the same analysis to the median gene expression levels of the NS genes and further examined the differences in these median levels between the PS and NS genes

We further investigated whether the yeast-strain signature that is pervasive between yeast strains and conditions of the single-cell organism (yeast) is also evolutionarily preserved in a multicellular organism (human). If it exists, we can hypothesize that the cancer cell lines with the growth (survival) preferring gene expression program should be more sensitive (resistant) to anticancer drug treatments. To test this, we first obtained cancer cell gene expression profiles and the drug response profiles of the corresponding cell lines. Then we obtained human orthologs for the PS and NS genes of yeast [34] and labeled human PS and NS genes, respectively (see the “Methods” section). The GO enrichment analysis result of the human PS and NS genes was also similar to that of yeast (Additional file 2: Table S6). Next, we calculated the median gene expression levels of the human PS and NS genes for each cell line, which we denote PS and NS scores per cell line, respectively. Then, we computed the correlation between the PS (and NS) score and the drug resistance across cancer cell lines and found that NS scores tend to show a positive correlation to the drug resistance for both GDSC1 and GDSC2 datasets [38, 39] (Fig. 6), indicating that the cell lines with relatively higher expression of the NS genes exhibit more resistance to various anticancer drugs. Further, the difference between PS score and NS score showed a stronger negative correlation with drug resistance (Fig. 6), suggesting that cell lines with a gene expression program that prefers growth and exhibits less stress resistance demonstrate less resistance to anticancer drugs.

**Fig. 7:**
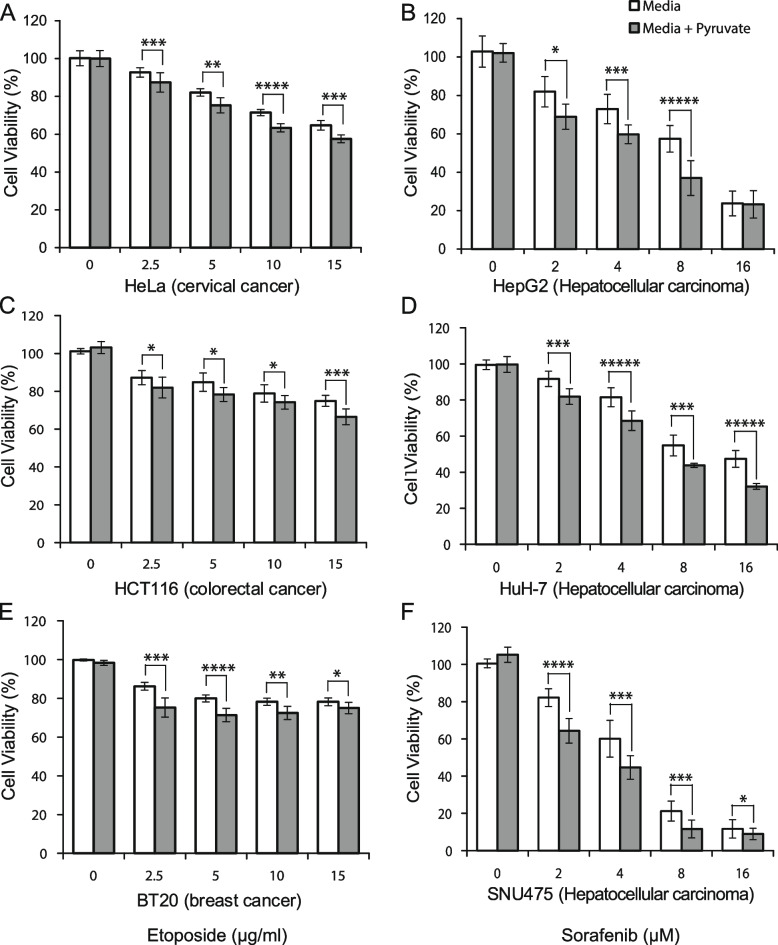
Decreased drug resistance by induced growth preference. Cells were cultured in high glucose (25 mM) DMEM for 24 h and then cultured in low-glucose (5.56 mM) DMEM with or without 1 mM sodium pyruvate. After 24 h, the cells were treated with the indicated concentrations of etoposide (sorafenib) and the controls were treated with the vehicle (DMSO) for 48 h. Cell viability was determined using the absorbance value of WST-8. Cell viability assays were performed in quadruplicate and were repeated three times (*p < 0.05, **p < 0.01, ***p < 0.005, ****p < 0.001, *****p < 0.0005; Student’s *t*-test). HCC indicates hepatocarcinoma

Based on this finding, we further investigated whether we could induce cancer cells to be more sensitive to anticancer drugs across various cancer cell lines. Pyruvate is one of the central nutrients that induce cellular growth by inducing mitochondrial oxygen consumption [40]. We compared the drug sensitivities in cancer cells in the presence and absence of pyruvate. We found that cancer cells with pyruvate treatment showed decreased cell viability, in other words, increased anticancer drug sensitivity, compared to the control when treated with the anticancer drug (Fig. 7). Importantly, we found that the increased drug sensitivity caused by pyruvate treatment did not depend on the type of cancer cells (hepatocellular carcinoma, colorectal cancer, breast cancer, or cervical cancer), mutational backgrounds (P53, RAS, Axin, etc.) nor the type of the anticancer drugs (multi-kinase inhibitor; sorafenib or cytotoxic drug; etoposide). Overall, we conclude that the molecular characteristics determining growth or survival in yeast also exist in human cells, and they can be utilized for anticancer drug treatment strategies.

## Discussion

The fitness trade-off, a phenomenon that arises when the fitness benefit in one aspect raises the fitness cost in another aspect, provides us with a useful conceptual framework to explain many biological processes, such as aging [41] or genetic disease [42]. A biological system should perform multiple, often opposing, tasks depending on the given conditions [43, 44]. Thus, the fitness trade-off is a common phenomenon, as a biological system with the best performance in one condition might not be able to obtain the best performance in another condition. This has also been observed between vegetative growth and resistance to microbial infection among natural variants of *Arabidopsis thaliana* [45], which suggests that the fitness trade-off is prevalent among natural variants.

Numerous studies have investigated that antagonistic pleiotropy where a single gene regulates more than one trait, and those traits have opposing effects on an organism’s fitness, is an underlying mechanism of the fitness trade-offs [42, 46]. A recent study has also shown that the variability of pleiotropic effects may significantly vary depending on the evolutionary trajectories and the specific conditions under which a yeast population evolves [47]. Another study found that clones which gained fitness in one environment tended to lose more fitness under conditions that were more different from the environment in which they evolved, suggesting the pervasive nature of the antagonistic pleiotropy in the evolution of yeast [48].

In this study, we found that natural yeast variants extensively demonstrate the fitness trade-off between growth and survival. A previous study [12] found that wild yeast strains generally have higher fitness under most stress conditions. Our analysis additionally revealed that these conditions are primarily nutrient-limiting and tend to display positively correlated phenotypes with each other (Additional file 2: Table S1). This suggests an adaptation in wild yeast strains primarily to starvation, with a fitness cost under other stress conditions. Comparing ESR, UGRR, and metabolic cycle gene expression between wild and domesticated strains, we observed a greater fold change in ESR-induced genes in wild strains, indicating higher expression compared to domesticated strains and supporting superior fitness in stressful environments (Additional file 1: Fig. S8). However, UGRR gene expression differences were not significant, implying that GRR gene expression is not a major factor distinguishing wild from domesticated strains. Interestingly, RB genes were more expressed in wild strains, implying a longer low oxygen consumption phase before cell cycle initiation compared to domesticated strains. Additionally, we found that domesticated strains exhibited clearer fitness trade-offs across various environmental stresses than wild strains (Additional file 1: Fig. S2 and S3). This pattern may be a result of domestication, as domesticated strains evolved in more uniform environments, contrasting with the variable conditions faced by wild strains. Consequently, a broader fitness trade-off or reduced trait complexity in domesticated yeast strains may reflect their evolution in less varied environments.

It is intriguing that IRA1 and IRA2, and the human orthologous NF1 (neurofibromin 1) genes, which both negatively regulate RAS activity, thereby regulating the balance between growth and survival, are all hypermutable genes [49]. For humans, the human ortholog NF1 has been shown that the gene is associated with multiple diseases, including cancer [50]. These genes are highly prone to mutation and can cause a reversible phenotypic switching [51], which suggests that their hypermutability can result in diverse phenotypic variations among individuals and provide fitness switching among yeast populations. This offers an overall fitness advantage to a population, as a subpopulation can easily emerge with the best fitness under a given condition. Therefore, the selection of hypermutability for IRA1 and IRA2 also supports the notion that they are strongly correlated with fitness trade-offs.

The slow growth rate and stress resistance within isogenic populations are well-known antagonistic phenomena in yeast [52] and *Caenorhabditis elegans* [53]. This indicates that the balance between growth preference and stress resistance can be triggered by stochastic fluctuation, aging, or environmental challenges as well as by genomic variation. We also found a similar phenomenon in cancer cells, as we showed that a cancer cell grown with pyruvate treatment has lower fitness when treated with an anticancer drug. On the other hand, the fitness advantage for the cancer cell following pyruvate treatment was not significant (Fig. 7). This might be because every cancer cell already obtained the maximal fitness advantage, and no additional fitness advantages could be obtained [54]. It should also be considered that the induced drug sensitivity observed in cancer cells treated with pyruvate might arise from confounding factors due to its significant effects on cancer metabolism [55], rather than solely from the mechanism we suggested. However, despite this, our proposed principle may still be useful for developing a new cancer therapeutic strategy without incurring additional side effects.

## Conclusions

In summary, we identified that the fitness trade-off between growth and stress resistance is the major determinant of the phenotypic variation within yeast strains. We further found that the same principle also holds for cancer cells. Together, we conclude that this might be a fundamental principle that explains the phenotypic differences among individuals.

## Methods

### Growth rate profiles

We obtained the following five yeast phenome datasets: 1011 natural strains under different nutritional sources [12]; 971 natural strains under 35 stress conditions [5]; 38 natural strains under 199 diverse conditions [56]; 52 natural strains under 13 stress conditions [57]; 1004 yeast segregants from a cross between BY and RM yeast under 46 diverse conditions [58]. For [56], we used “growth efficiency,” total change in population density for 72 h [56], as a growth phenotype for our analysis, but we also found that our conclusion largely remains consistent even if we apply other growth phenotypes (Additional file 1: Fig. S4). We removed the rows or columns of which more than 10% of values were missing. Then, the missing values were imputed using knnimpute [59] function in R. Then, we performed sample-wise normalization with a mean of 0 and a variance of 1 for each growth condition. Next, we performed singular value decomposition (SVD) analysis for the phenome matrix* M*, as follows:

$$M=U*S* {V}^{T}$$

where the columns of* U* are left-singular vectors that are orthogonal with each other, *S* is a diagonal matrix, and the columns of *V* are right-singular vectors. Then, *M* can be reconstructed by the following matrix multiplication:

$$M={\sum }_{k=1}^{n}{S}_{k}*{U}_{k}* {{V}_{k}}^{T}$$

where *n* indicates the number of yeast strains for each phenome dataset. We used a reconstructed ${M}_{R}$ using the first two most significant ranks (*n* = *2*) instead of the original *M* to exclude the effect of growth phenotypes that are extremely specific to a certain condition (Fig. 1). We also performed the same analysis using the raw phenome datasets and found consistent results except one dataset (Additional file 1: Fig. S1). We used the svd function in R.

### Consensus clustering analysis

For each phenome dataset, we performed hierarchical clustering analysis using 1 − Spearman rank correlation as a distance measure and applied average linkage method. To obtain the optimal number of clusters, we performed consensus clustering analysis using ConsensusClusterPlus [60] in R. Clusters were obtained through 1000 resampling iterations of the hierarchical clustering by randomly selecting 80% of the samples and 80% of features. Then, we obtained a condition-to-condition consensus matrix for each number of cluster and computed the proportion of ambiguous clustering (PAC) [61] which denotes the ratio of condition pairs that are ambiguously co-clustered between all pairs. If two conditions are always clustered together, the consensus index value of this pair is equal to 1; if they never be clustered together, the value is 0. Then, we have defined that a pair of conditions are ambiguously co-clustered if its consensus index value is between 0.1 and 0.9 out of 1000 resampled iterations. Therefore, for a given cluster number *K*, the PAC is computed as the proportion of ambiguous pairs, and the lower PAC indicates the more robust cluster.

### mRNA expression profiles

For Agilent microarray datasets in batch cultures, we obtained the following four yeast inter-strain gene expression profiles: 121 yeast segregants of a cross between BY and RM under ethanol and glucose treatments (GSE9376) [14]; 6 yeast strains (Ds288c, EM93, Sgu52, Sgu407, Sgu421, and Sg60) in four different environments (GSE3021) [15]; 196 yeast segregants from two sets of segregants of crosses: DBY8268 and M22, and DBY8268 and YPS163 (GSE54196) [13] under ethanol treatment. We removed the rows or columns of which more than 10% of values were missing, and the missing values were imputed by knnimpute [59] in R and sample-wise zero-center scaled. For the RNA sequencing dataset in batch cultures, we analyzed RNA-seq data from natural yeast isolates [16] grown in standard synthetic complete media with 2% glucose as the carbon source. This analysis focused on strains labeled as “wild,” “unassigned,” or “domesticated (dom),” yielding a total of 943 samples. We obtained the raw count matrix, then applied variance stabilizing transformations using vst function in the DEseq2 package [62] in R. For the Affymetrix microarray datasets in continuous cultures, we obtained four distinct sets of yeast gene expression profiles: 12 gene expression profiles of a laboratory yeast strain CEN.PK113-7D grown at steady state in aerobic continuous cultures on a minimal medium with glucose as the limiting nutrient (E-MEXP-593) [20]; 36 gene expression profiles from the same laboratory yeast strain under multiple conditions, varying in dilution rate, nutrient limitation, and oxygen availability (E-MTAB-78) [45]; 170 profiles from the same strain across different conditions, utilizing multiple sample preparation protocols, with normalized expression profiles sourced from the supplementary files (12864_2008_1937_MOESM6_ESM.zip) (GSE11452) [18]; 48 gene expression profiles from yeast strain FY1679 subjected to multiple nutrient-limiting conditions (E-MEXP-115) [17].

The Agilent microarray datasets from batch cultures are measured based on a two-color (Cy5 and Cy3) hybridization protocol of a scanning system, where one color is from a sample RNA and the other is from a reference, a pooled RNA. Therefore, the value for each gene denotes the relative expression level of that gene in the sample compared to the averaged expression of the gene across all samples. On the other hand, each value denotes absolute expression levels for RNA sequencing or Affymetrix microarray. Therefore, we standardized these datasets to the same scale as the hybridization system, we normalized the expression level of each gene by subtracting the gene-wise mean value and then applied sample-wise zero-center scaling.

### Finding a recurrent gene expression signature

First, we performed SVD analysis for each of the sample-wise scaled gene expression matrices. A previous study [63] defined eigengenes and eigenarray, where eigengenes and eigenarrays are the vector in each row of the matrix ${V}^{T}$ and the vector in each column of the matrix U, respectively. Then, the eigengenes represent the activities of independent regulatory gene expression programs and the eigenarrays represent the corresponding cellular states. In our analysis, we focused on the first eigenarray—the left-most column of U—which represents the most influential cellular state underlying the actual cellular states of yeast strains. Thus, the first column of U is a vector containing yeast genes, each with a specific value. Then, we obtained the first column of U for each matrix, which we call a “recurrent gene expression signature.” Next, we integrated the gene expression datasets of Agilent and Affymetrix microarrays, respectively. We selected the genes that are present in all the datasets and batch-corrected using the ComBat function in R [64]. The resulting integrated microarray matrices in both datasets are composed of 6029 genes and 493 samples, and 6057 genes and 266 samples, respectively. RNA sequencing dataset across yeast strains is composed of 6517 genes and 943 samples. To obtain robust recurrent gene expression signature for each integrated dataset, we conducted SVD analysis iteratively 1000 times, adding Gaussian noise (mean = 0 and standard deviation = 1) to each gene expression value. We then selected only those genes that consistently exhibited the same sign across all iterations. This approach allows us to obtain robust and recurrent gene expression signatures, resulting in two yeast strain signatures (Agilent microarray and RNA-seq) and one slow-growth signature (Affymetrix microarray) (Fig. 3). The three robust recurrent gene expression signatures are listed in Additional file 2: Table S2. For the two yeast-strain signatures, we further filtered the genes with the same sign and obtained an averaged gene signature score for each gene, then obtained an integrated yeast-strain signature (Additional file 2: Table S2).

### Identifying the largest connected component

We downloaded a network dataset from STRING [65], version 11.5. This network consists of nodes and links, where nodes represent genes and links denote the functional interaction scores between pairs of genes. These scores range from 0 to 1, and we considered only links with a score higher than 0.4, indicating medium confidence. Using the *p*-values from the GWA analysis, we determined the *p*-value for each gene by selecting the lowest *p*-value among the loci intersecting with the gene body. After selecting specific genes (for example, the 50 genes with the lowest *p*-values), we extracted the subnetwork and computed the size of its largest connected component. For comparison, we did the same calculation from randomly selected genes with the same number.

### Finding human orthologs from yeast/human orthologs

We downloaded yeast/human orthologs from the *Saccharomyces* Genome Database (SGD) [34]. From the PS and NS gene sets, we obtained corresponding human PS and NS genes, respectively. The number of human PS and NS genes is 297 and 77, respectively.

### Genetic measurements

We obtained UGRR genes [22], ESR genes [21], and metabolic cycle genes [23]. Haploid fitness was obtained based on growth rates for haploid knockout (KO) for each gene [26]. Stochastic noise for each gene was measured using proteomic abundance, and the stochastic noise was computed as the coefficient of variation to the distance-to-median for each gene [27, 28]. We also obtained mRNA half-life and mRNA abundance variability which was computed as the standard deviation of the mRNA abundances across different conditions [30].

### Computing the association between genotype and growth phenotype

We performed principal component analysis (PCA) analysis on the growth rate dataset and obtained PC1 values as an overall growth rate for each strain [11]. We calculated LOD (logarithm of the odds) scores for each genotypic marker and the overall growth rate as $- n(\ln (1 - r^{2} )/2(\ln (10))$, where *n* denotes the number of segregants (in this case, *n* = 1008) and *r* indicates the Pearson correlation coefficient between the segregant genotypes and trait values. The source code for this computation was adopted from [58]. For GWA analysis, we used factored spectrally transformed linear mixed models (FaST-LMM) [66]. We downloaded a file named 1011GWASMatrix.tar.gz for the genotype dataset, and phenoMatrix_35ConditionsNormalizedByYPD.tab.gz for the growth phenotype across 971 yeast strains under 35 different conditions [5]. Then we computed PC1 values based on the growth phenotype matrix and performed FaST-LMM using the PC1 values for each strain as a representative phenotype. The datasets are at http://1002genomes.u-strasbg.fr/files/ and details are described in [5].

### Anticancer drug responses and gene expression profiles of human cancer cell lines

We downloaded GDSC1 and GDSC2 [38, 39]. From the datasets, we used “Z_SCORE”, which is a normalized IC50 value for each drug across cancer cell lines as a drug resistance measure for each anticancer drug and cancer cell line pairs. A higher Z_SCORE indicates greater resistance of the drug to the targeted cancer cell line. We also downloaded the cancer cell gene expression profiles from the Cancer Cell Line Encyclopedia (CCLE) [67] (ndownloader.figshare.com/files/34989919). We normalized the expression level of each gene by subtracting the gene-wise mean value. To obtain human PS and NS gene scores, we computed the median gene expression level of the human PS and NS genes, respectively, for each cell line. Then, we computed the Spearman’s rank correlation between the drug resistance and PS (or NS) gene scores for each anticancer drug, respectively. We further computed the difference between PS and NS gene scores and performed the same analysis.

### Cell culture

BT20, HCT116, HeLa, HepG2, Huh-7, and SNU475 cells were obtained from Korean Cell Line Bank (KCBL) and were cultured in high glucose (25 mM) Dulbecco’s modified Eagle’s medium (DMEM) supplemented with 10% fetal bovine serum (FBS) and antibiotics (Life Technologies Corp. Carlsbad, CA, USA) at 37 °C in a humidified atmosphere containing 5% CO_2_.

### Cell viability assays

For the cell viability assays, the cells were seeded in 96-well plates at a density of 5000 cells/well (100 μL total volume/well) and were grown for 24 h. The cells were then washed with sterile phosphate-buffered saline and maintained in low-glucose (5.56 mM) DMEM supplemented with 10% FBS with/without sodium pyruvate (1 mM, Thermo Fisher Scientific, Waltham, MA, USA) for 24 h before adding the sorafenib (LC Laboratories, Woburn, MA, USA) or etoposide (Sigma-Aldrich, Saint Louis, MO, USA). The cells were treated with different concentrations of sorafenib or etoposide, and the controls were treated with vehicle (DMSO, Sigma-Aldrich). After 24-h treatment, WST-1 solution (DoGenBio, Seoul, South Korea) was added to the cells for 1–2 h, and the absorbance at 450 nm was then measured using a VICTORTMX3 Multilabel Plate Reader (PerkinElmer Inc., Waltham, MA, USA).

### Supplementary Information

**Additional file 1: Figure S1–S8**. **FigS1.** The fitness trade-off under various environmental conditions. **FigS2.** The clustering results of the growth phenotype under various nutritional conditions (Fig. 1A) for each yeast clade.** FigS3.-** The clustering results of the growth phenotype under various stress conditions (Fig. 1B) for each yeast clade. **FigS4.** The clustering results of the growth phenotype under various environmental conditions (Fig. 1C) for each stress group (carbon utilization, environment & metabolites, nitrogen utilization, nutrient requirements and toxins) and growth phenotype measurement (growth efficiency, growth rate, and growth lag).  **FigS5.** A recurrent molecular signature across various gene expression profiles of yeast. **FigS6.** Scatter plots between the recurrent gene expression signature and various genetic measurements. **FigS7.** The association between the genotype and the growth phenotype across each yeast clade. **FigS8.-** The fold change (Log2) differences between wild and domesticated strains across each gene set.

**Additional file 2: Table S1–S6. Table S1. **Conditions forming clusters for each dataset. **Table S2.** Recurrent gene signature and the signature score. **Table S3.** The GO analysis for PS and NS genes in yeast. **Table S4.** The GO analysis for yeast-strain specific PS and NS genes in yeast. **Table S5.** GO analysis for genes comprising the largest connected component. **Table S6.** The GO analysis for PS and NS genes in human.

**Additional file 3. **R script and datasets used for generating the figures and tables.

## Data Availability

All the datasets analyzed in this study are publicly available. The source code for the analysis is uploaded as supplementary data.

## Abbreviations

QTL: Quantitative trait loci
GWA: Genome-wide association
ESR: Environmental stress response
UGRR: Universal growth-rate response
RC: Reductive charging
OX: Oxidative
RB: Reductive building
PS genes: Positive-scored genes
NS genes: Negative-scored genes
GO: Gene ontology
GDSC: Genomics of drug sensitivity in cancer
SVD: Singular value decomposition
PAC: Proportion of ambiguous clustering
SGD: *Saccharomyces* Genome Database
PCA: Principal component analysis
LOD: Logarithm of the odds
FAST-LMM: Factored spectrally transformed linear mixed models
IC50: Half maximal inhibitory concentration
CCLE: Cancer Cell Line Encyclopedia
KCLB: Korean Cell Line Bank
DMEM: Dulbecco’s modified Eagle’s medium
FBS: Fetal bovine serum
DMSO: Dimethyl sulfoxide
