# Supplementary material for: The fitness trade-off between growth and stress resistance determines the phenotypic landscape
Source: BMC Biol. 2024 Mar 13;22:62. doi: 10.1186/s12915-024-01856-7 (PMC10935846; doi:10.1186/s12915-024-01856-7)
Supplement: Supplementary file 1 — Additional file 1: Figure S1–S8. FigS1. The fitness trade-off under various environmental conditions. FigS2. The clustering results of the growth phenotype under various nutritional conditions (Fig. 1A) for each yeast clade. FigS3.- The clustering results of the growth phenotype under various stress conditions (Fig. 1B) for each yeast clade. FigS4. The clustering results of the growth phenotype under various environmental conditions (Fig. 1C) for each stress group (carbon utilization, environment & metabolites, nitrogen utilization, nutrient requirements and toxins) and growth phenotype measurement (growth efficiency, growth rate, and growth lag). FigS5. A recurrent molecular signature across various gene expression profiles of yeast. FigS6. Scatter plots between the recurrent gene expression signature and various genetic measurements. FigS7. The association between the genotype and the growth phenotype across each yeast clade. FigS8.- The fold change (Log2) differences between wild and domesticated strains across each gene set. [file 12915_2024_1856_MOESM1_ESM.docx]

**
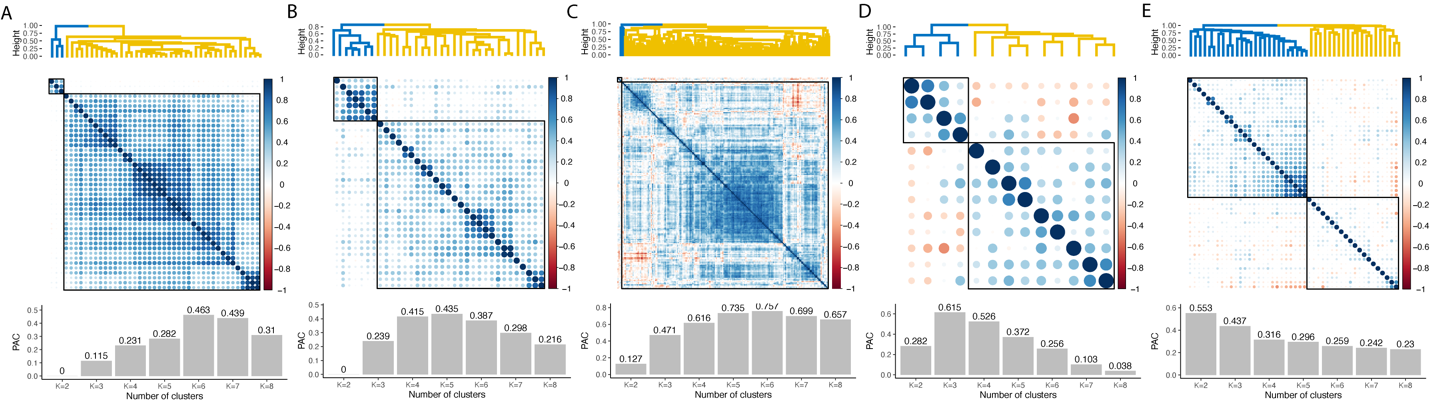
**

**Fig S1. The fitness trade-off under various environmental conditions.** We performed the same analysis using the raw phenome datasets used in Fig. 1. We note that (A) and (C) show two optimal clusters. In (B), three clusters are the optimal number of clusters, however, if we consider one extreme condition (CuSO_4_) as an outlier, the same conditions were considered as in Fig. 1B. In (D), there are only 13 conditions to be clustered, so two clusters are the optimal number of clusters without singletons. (E) is the only exception that does not provide two robust clusters.

**
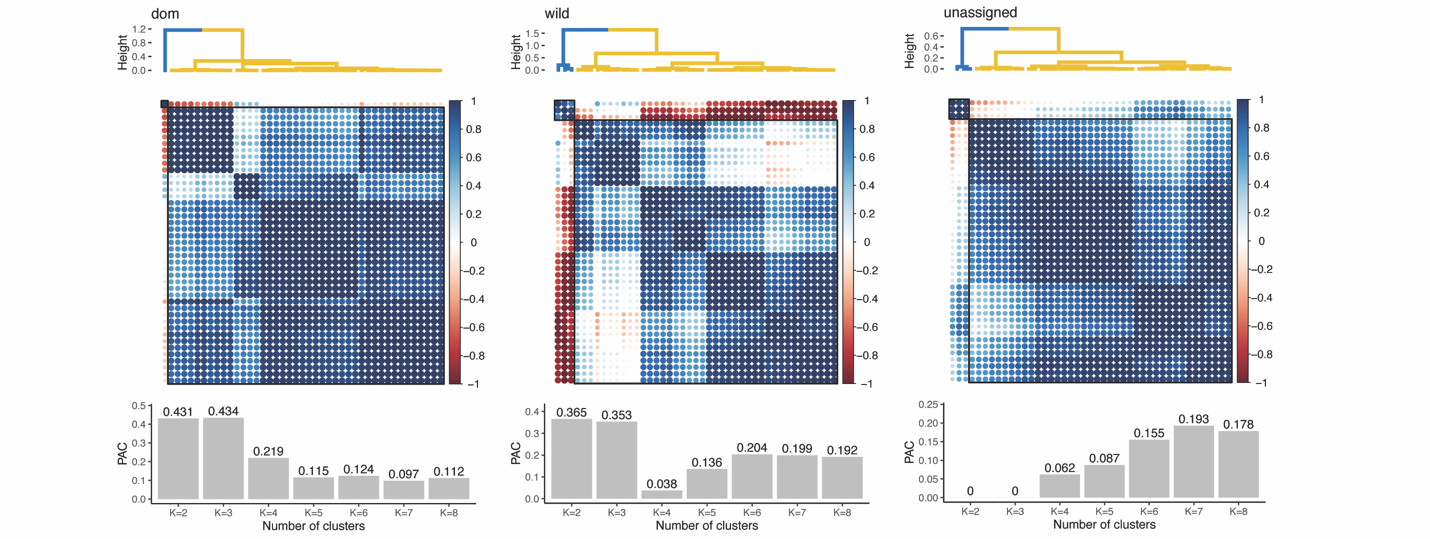
**

**Fig. S2. The clustering results of the growth phenotype under various nutritional conditions (Fig. 1A) for each yeast clade (1).** ‘dom’ denotes domesticated strain.

**
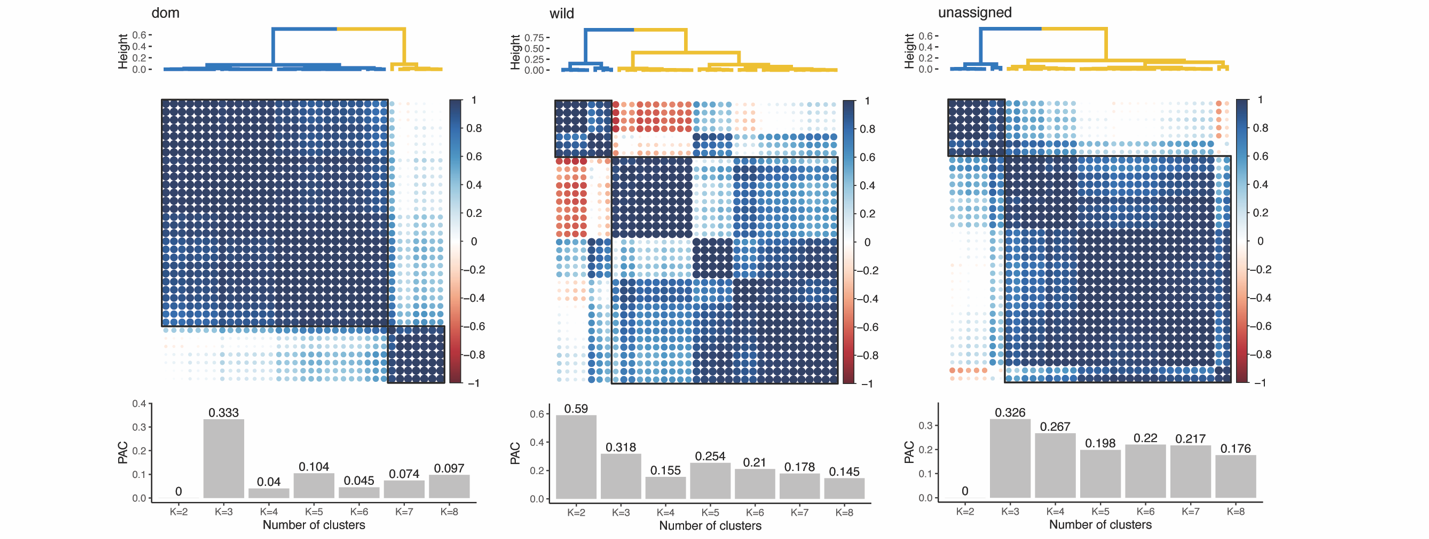
**

**Fig. S3. The clustering results of the growth phenotype under various stress conditions (Fig. 1B) for each yeast clade (2).** ‘dom’ denotes domesticated strain.

**
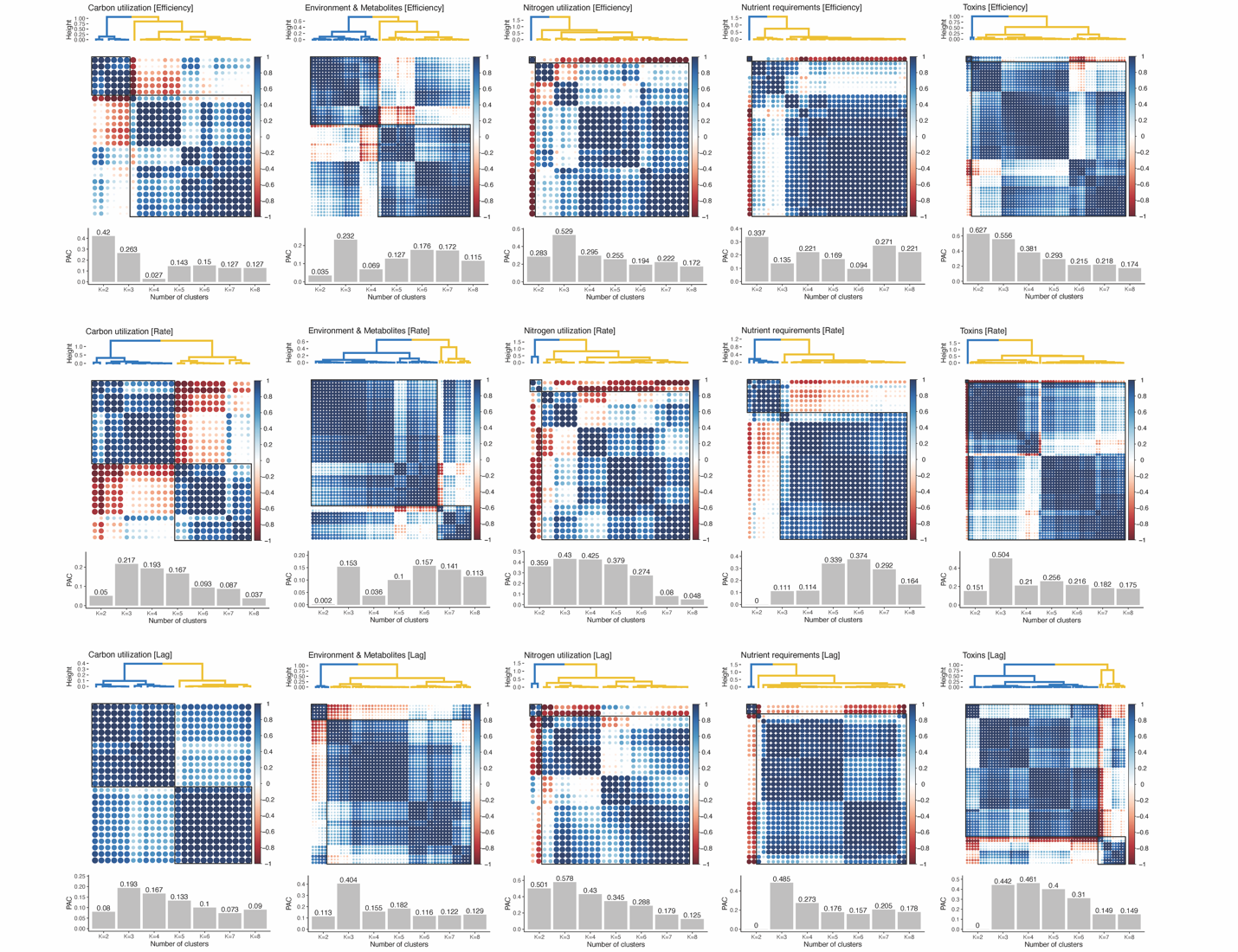
**

**Fig. S4. The clustering results of the growth phenotype under various environmental conditions (Fig. 1C) for each stress group (carbon utilization, environment & metabolites, nitrogen utilization, nutrient requirements and toxins) and growth phenotype measurement (growth efficiency, growth rate and growth lag) (3).** We have taken into consideration the observations that growth lag, as measured by population adaptation time, has no correlation to the other fitness-associated phenotypes, growth efficiency and growth rate showing strong correlation with one another (3). This suggests that the growth lag may be regulated by mechanisms that are independent of those controlling growth efficiency and growth rate. Therefore, the two-cluster formation under the conditions with toxins for growth lag phenotype is unlikely to be related to the fitness trade-off raised in this study. Furthermore, growth efficiency and growth rate phenotypes (after excluding one outlier condition, arabinose 2%, for growth rate) with toxins do not split into two clustered conditions (data not shown). Overall, we can summarize that this observation with toxins does not contradict our main conclusion regarding the fitness trade-off between growth and stress resistance.

**
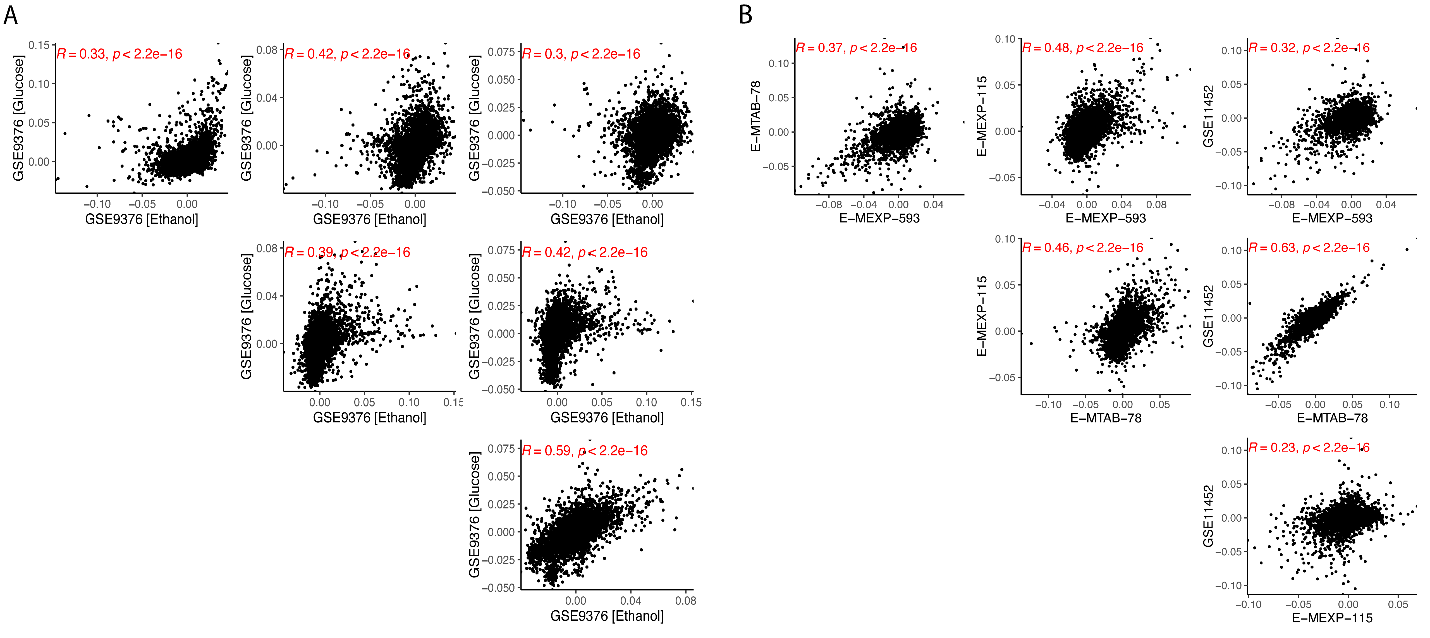
**

**Fig. S5. A recurrent molecular signature across various gene expression profiles of yeast.** Scatter plots for the recurrent gene expression signatures between two gene expression profiles of yeast. (A) The recurrent gene expression signature of various yeast strains under various conditions. GSE9376 (Glucose) and GSE9376 (Ethanol) denote the gene expression profiles of 109 segregants from BY and RM under glucose and ethanol treatment, respectively. GSE3021 indicates six strains grown in four different environments representing a continuum of rich and poor natural conditions. GSE54196 denotes the gene expression response to ethanol stress for segregant strains from two crosses: DBY8268 x M22 and DBY8268 x YPS163. (B) The recurrent gene expression signature of laboratory yeast strains under nutrient-limiting but non-stress conditions. The gene expression profiles are from the lab yeast strain CEN.PK113-7D in nutrient-limited, non-stress conditions, including steady-state growth in glucose-limited media (E-MEXP-593), varying conditions of dilution and oxygen (E-MTAB-78), and different conditions and protocols (GSE11452). E-MEXP-115 is the gene expression profiles from FY1679 yeast strains subjected to multiple nutrient-limiting environments. R and p-value denote Spearman’s rank coefficient and the corresponding p-value.


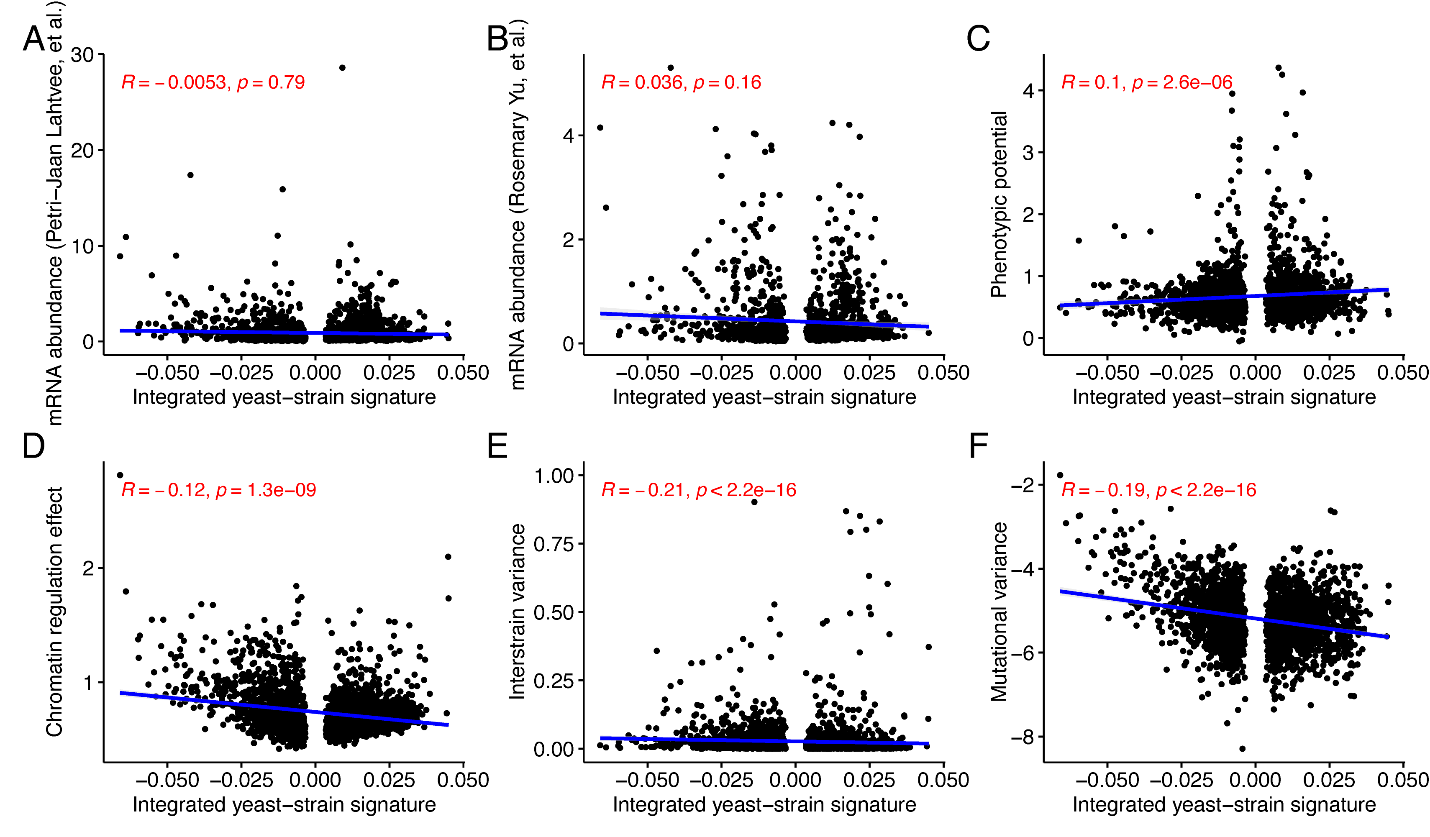


**Fig. S6. Scatter plots between the recurrent gene expression signature and various genetic measurements.** The recurrent gene expression signature was obtained from the first column of U by SVD. (A) The median absolute mRNA abundances under 10 different conditions were obtained from (4). (B) The median absolute mRNA abundances under 27 different nitrogen metabolites (NM) conditions were obtained from (5). (C) The phenotypic potential is the degree of phenotypic variability across 70 morphological phenotypes when a gene is deleted (6). (D-F) Interstrain variance, chromatin regulation effect, and mutational variance were obtained from the literature (7). For short, interstrain variance denotes gene expression variability across yeast strains, chromatin regulation effect indicates gene expression variability upon deletion of various chromatin regulators, and mutational variance means the gene expression variability upon spontaneous mutations of a yeast strain.


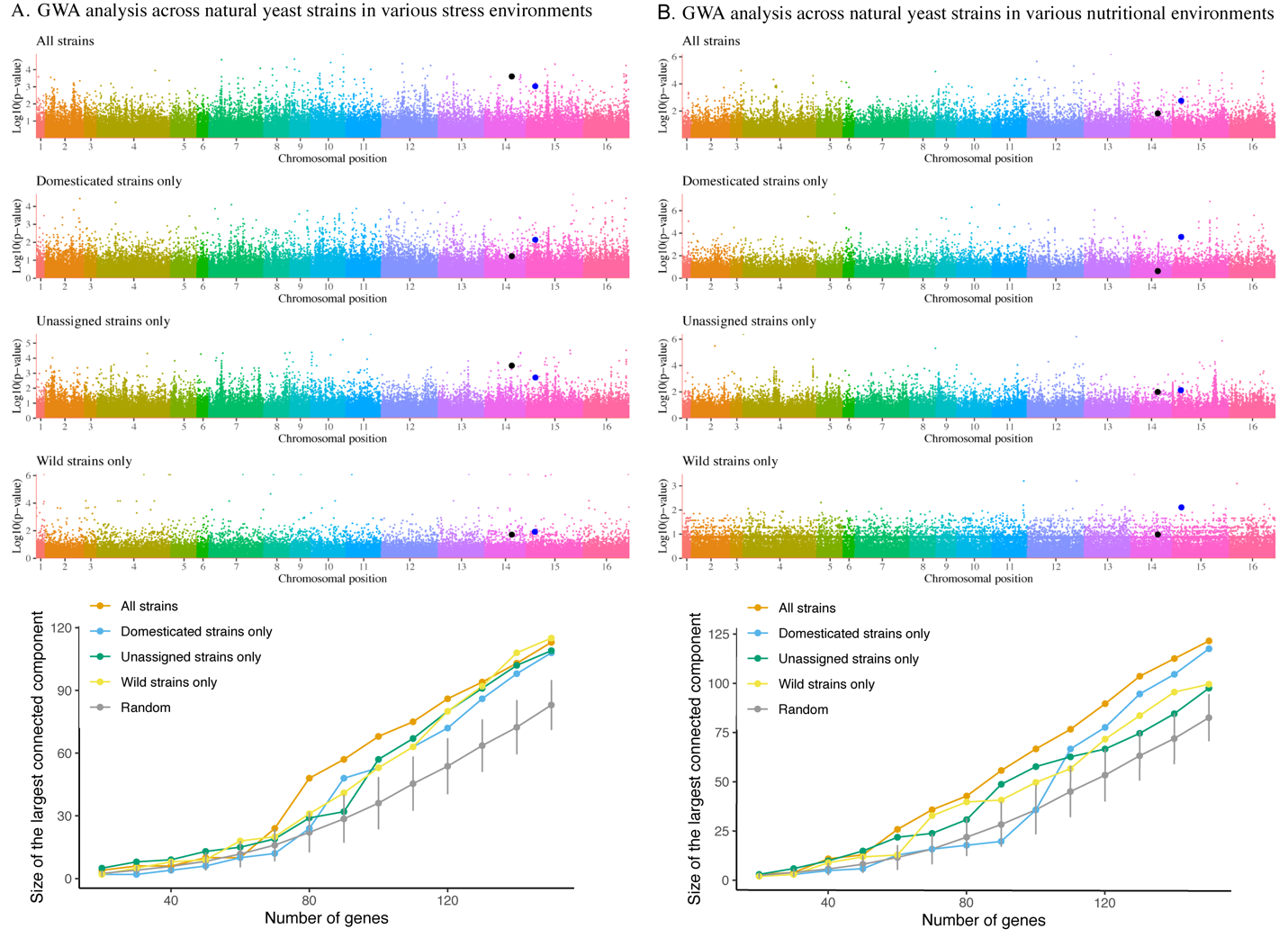


**Fig. S7. The association between the genotype and the growth phenotype across each yeast clade.** Manhattan plots depict GWA between the overall growth phenotypes under various nutritional and stress conditions, and the genotypes of different yeast strains. Error bar plots depict the network size of the top-ranked genes and the random gene samples with the same number. The x-axis represents the number of genes analyzed, while the y-axis shows the size of the largest connected component in the gene interaction networks. (A and B) GWA analysis across yeast strains for stress and nutritional conditions. For ‘All strains’, the figures are the same to the Fig. 4B and C. Black and blue dots indicate the loci of MKT1 and IRA2 genes, respectively.

**
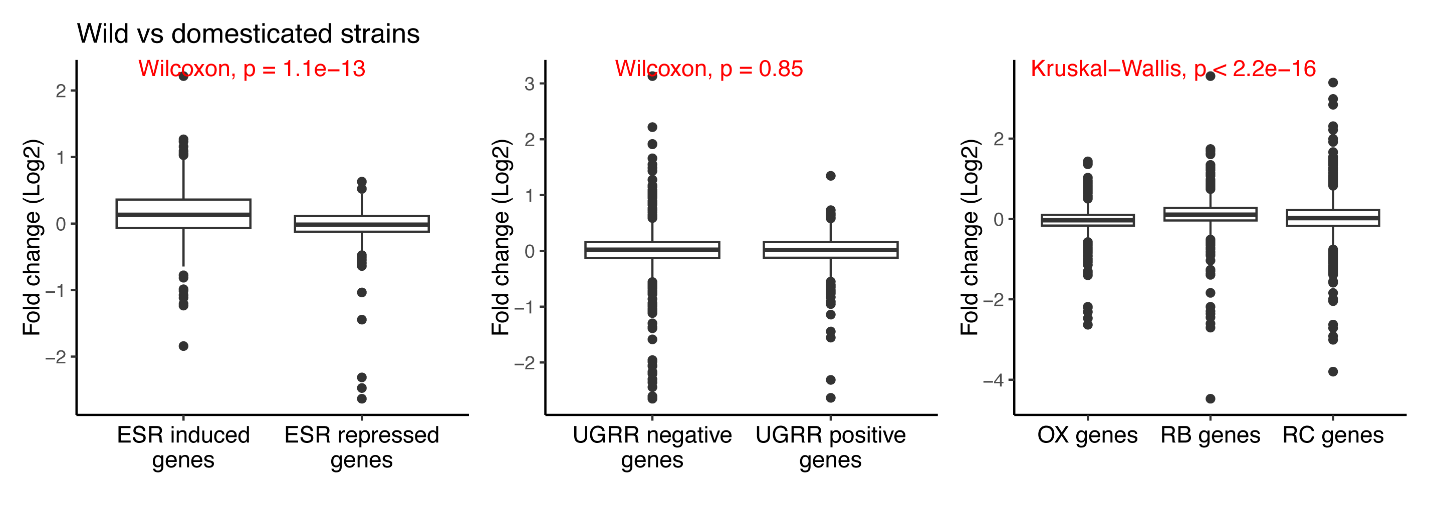
Fig. S8. The Fold change (Log2) differences between wild and domesticated strains across each gene set.** Positive (or negative) fold change indicates the gene is more expressed in wild (or domesticated) strains than the other strains. For pairwise comparison, Wilcoxon’s rank sum test was performed. For comparing three gene sets, Kruskal–Wallis test was used.

**References**

1. De Chiara,M., Barré,B.P., Persson,K., Irizar,A., Vischioni,C., Khaiwal,S., Stenberg,S., Amadi,O.C., Žun,G., Doberšek,K., *et al.* (2022) Domestication reprogrammed the budding yeast life cycle. *Nat Ecol Evol*, **6**, 448–460.

2. Peter,J., De Chiara,M., Friedrich,A., Yue,J.-X., Pflieger,D., Bergström,A., Sigwalt,A., Barre,B., Freel,K., Llored,A., *et al.* (2018) Genome evolution across 1,011 Saccharomyces cerevisiae isolates. *Nature*, **556**, 339–344.

3. Warringer,J., Zörgö,E., Cubillos,F.A., Zia,A., Gjuvsland,A., Simpson,J.T., Forsmark,A., Durbin,R., Omholt,S.W., Louis,E.J., *et al.* (2011) Trait variation in yeast is defined by population history. *PLoS Genet*, **7**, e1002111.

4. Lahtvee,P.-J., Sánchez,B.J., Smialowska,A., Kasvandik,S., Elsemman,I.E., Gatto,F. and Nielsen,J. (2017) Absolute Quantification of Protein and mRNA Abundances Demonstrate Variability in Gene-Specific Translation Efficiency in Yeast. *Cell Systems*, **4**, 495-504.e5.

5. Yu,R., Vorontsov,E., Sihlbom,C. and Nielsen,J. (2021) Quantifying absolute gene expression profiles reveals distinct regulation of central carbon metabolism genes in yeast. *eLife*, **10**, e65722.

6. Levy,S.F. and Siegal,M.L. (2008) Network Hubs Buffer Environmental Variation in Saccharomyces cerevisiae. *PLoS Biol*, **6**, e264.

7. Choi,J.K. and Kim,Y.-J. (2009) Intrinsic variability of gene expression encoded in nucleosome positioning sequences. *Nat Genet*, **41**, 498–503.
